# Chronic Pain Acceptance Moderates the Association Between Pain Intensity and Alcohol Use Severity Among Veterans With Chronic Musculoskeletal Pain

**DOI:** 10.1111/acer.70305

**Published:** 2026-06-12

**Authors:** Kyle M. White, Victoria E. Carlin, Joon Kyung Nam, Joseph W. Ditre

**Affiliations:** ^1^ Center for Health Behavior Research & Innovation, College of Arts & Sciences Syracuse University Syracuse New York USA; ^2^ Department of Psychology, College of Arts & Sciences Syracuse University Syracuse New York USA; ^3^ D'Aniello Institute for Veterans & Military Affairs Syracuse University Syracuse New York USA

**Keywords:** alcohol, chronic pain acceptance, pain, veterans

## Abstract

**Background:**

Chronic pain and hazardous alcohol use are prevalent and commonly co‐occur among US veterans. A growing literature highlights pain as a motivator of alcohol consumption, with evidence suggesting more intense pain is associated with an increased likelihood of drinking for pain management. Although chronic pain acceptance (i.e., willingness to experience chronic pain and its sequelae while maintaining engagement in valued life activities) has emerged as a protective factor in the context of opioid‐related behavior, its role in shaping pain‐alcohol relations has not been examined. The goal of this cross‐sectional study was to test chronic pain acceptance as a moderator of the association between pain intensity and alcohol use severity among veterans with chronic musculoskeletal pain.

**Methods:**

Veterans (*N* = 429; *M*_age_ = 56.6) were recruited via Qualtrics Panels for an online survey. Measures included the Graded Chronic Pain Scale, Chronic Pain Acceptance Questionnaire, and Alcohol Use Disorders Identification Test. Hierarchical linear regression and conditional effects models were used to test associations between pain intensity, chronic pain acceptance, and alcohol use severity.

**Results:**

Chronic pain acceptance moderated the relationship between pain intensity and alcohol use severity, with a positive association observed at low but not moderate or high levels of acceptance. Exploratory subgroup analyses among veterans scoring above threshold for hazardous drinking also revealed an interaction, though none of the conditional effects were statistically significant.

**Conclusions:**

The current findings suggest that higher levels of chronic pain acceptance may buffer the impact of pain on alcohol use. Additional research is needed to evaluate the utility of acceptance‐based interventions for veterans with co‐occurring chronic pain and hazardous drinking.

## Graphical abstract

This study examined whether chronic pain acceptance moderates the association between pain intensity and alcohol use severity among US veterans with chronic pain. Higher pain intensity was associated with greater alcohol use only among individuals with low levels of chronic pain acceptance, suggesting that acceptance may buffer pain‐related drinking risk.

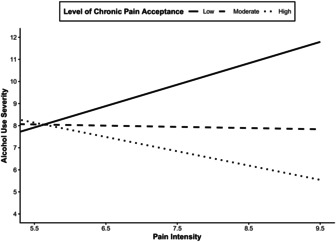

## 1 Introduction

Hazardous alcohol use (i.e., drinking behavior associated with increased risk of negative outcomes; MacKillop et al. 2022) represents a substantial public health concern among United States military veterans (Fuehrlein et al. 2016), with evidence from a nationally representative cohort that more than one in four exhibit patterns of hazardous drinking (Na et al. 2023). Chronic pain (i.e., pain that persists or recurs for more than three months; Treede et al. 2019) affects approximately 32% of veterans (Zelaya et al. 2020), who utilize more health care, experience higher rates of physical and mental comorbidities, and endorse lower quality of life when compared to veterans without pain (Forman‐Hoffman et al. 2007; Harding et al. 2019; Higgins et al. 2014). The co‐occurrence of chronic pain and hazardous alcohol use is common in veteran samples and may contribute to poorer treatment outcomes (Loughran et al. 2025; Morasco et al. 2011; Vowles et al. 2022).

Pain and alcohol use are proposed to have a reciprocal relationship, leading to an escalation in both over time (Ditre et al. 2019; Zale et al. 2015). For example, up to 50% of individuals seeking treatment for hazardous alcohol use experience recurrent pain (Boissoneault et al. 2019). Experimental findings indicate that pain induction increases self‐reported urge and intention to drink (Moskal et al. 2018), as well as ad‐lib alcohol consumption (Ditre et al. 2023). Additionally, higher pain intensity has been associated with an increased likelihood of drinking for pain management. Neurobiological evidence suggests chronic pain and alcohol dependence share overlapping neural substrates, with allostatic load (e.g., from repeated episodes of alcohol intoxication and withdrawal) contributing to dysregulation of reward and stress circuitry underlying both conditions (Egli et al. 2012).

Chronic pain acceptance (i.e., willingness to experience chronic pain and its sequelae while maintaining engagement in valued life activities; Reneman et al. 2010) has received empirical attention as playing an important role in pain‐related behaviors and outcomes (Kim and Kratz 2021; McCracken and Vowles 2006; Thompson and McCracken 2011). Although the inclination to avoid pain is natural (Walters and Williams 2019), pervasive, rigid, and habitual attempts to control pain can increase pain‐related distress and contribute to maladaptive coping (McCracken and Vowles 2006; Thompson and McCracken 2011). The behavioral drive for immediate relief or reduction in physical and psychological discomfort has been described as “destructive experiential avoidance” (Feliu Soler et al. 2018; Hayes et al. 1996), reflecting avoidance strategies that are negatively reinforced (Fordyce 1984) and has been associated with reductions in pain tolerance and increases in pain intensity (Goubert et al. 2004; Masedo and Rosa Esteve 2007). Of note, unhealthy substance use behavior has also been conceptualized as a means of experiential avoidance (Hayes et al. 1996). Thus, the association between pain intensity and alcohol use may be stronger among individuals who struggle to accept their chronic pain. For example, meta‐analytic evidence indicates a moderately sized negative association between chronic pain acceptance and pain‐related disability (White et al. 2024). Additionally, prior research has found chronic pain acceptance to be negatively associated with opioid use severity and motivation to use opioids to cope with pain (Lin et al. 2015; Smit et al. 2023). Chronic pain acceptance has further been shown to moderate the relationship between pain intensity and dependence on pain medication, such that more intense pain is associated with greater dependence at low levels of pain acceptance but less dependence at high levels of pain acceptance (Elander et al. 2014). Given that alcohol confers acute analgesia and is also used for pain coping (Goebel et al. 2011; Riley and King 2009; Thompson et al. 2017), it stands to reason that chronic pain acceptance may serve as a protective factor that buffers against engaging in alcohol use when pain intensifies.

The US Department of Veterans Affairs (VA) has emphasized the importance of integrated treatments for comorbid medical conditions and substance‐related disorders in their guidelines for clinical practice (Perry et al. 2022). Additionally, national initiatives arising in response to the national opioid crisis have highlighted the need for safer approaches to managing chronic pain (Fletcher et al. 2018). These initiatives, and prior work indicating that nearly one in four veterans receiving outpatient care use alcohol for pain‐coping (Goebel et al. 2011), underscore the importance of examining malleable psychological factors (e.g., chronic pain acceptance) that may link pain and alcohol use among veterans. Given that alcohol confers acute analgesic effects (Thompson et al. 2017) and chronic pain acceptance has been shown to moderate the relationship between pain intensity and dependence on pain medication (Elander et al. 2014), it stands to reason that acceptance of chronic pain may attenuate the association between pain intensity and alcohol use severity. However, the moderating role of chronic pain acceptance in pain‐alcohol relations, to our knowledge, has not been tested.

The goal of this study was to test the hypothesis that chronic pain acceptance would moderate the relationship between pain intensity and alcohol use severity among veterans with chronic musculoskeletal pain such that the positive association between pain intensity and alcohol use severity would be stronger at lower levels of chronic pain acceptance. To further contextualize findings, we also explored whether the hypothesized moderation effect was evident among a subsample of veterans scoring above an established clinical threshold for hazardous alcohol use.

## 2 Method

### 2.1 Participants and Procedure

Participants included 429 veterans of the US Armed Forces who were recruited via Qualtrics Panels, an online survey platform that sources prospective participants from vetted third‐party panel providers based on study eligibility requirements. Online panel recruitment methodology has been employed in chronic pain and substance use research (Doorley et al. 2024), with evidence suggesting that prescreening procedures used by Qualtrics Panels yield higher quality samples than platforms drawing from larger, less‐targeted respondent pools (Ibarra et al. 2018). Prospective participants were emailed a link to a description of the study and pre‐survey screening items on Qualtrics, which assessed the following inclusion criteria: (1) 18+ years of age; (2) US residence; (3) chronic musculoskeletal pain (assessed using a single item, “Do you currently suffer from chronic musculoskeletal pain, that is, pain that persists or recurs for more than three months and is located in the muscles, bones, joints, or tendons (e.g., low back pain, neck pain, arthritis pain, etc.)?”); (4) past‐month alcohol use (assessed by a single item, “Have you consumed alcohol in the past month?”); and (5) US veteran status. Respondents were excluded if they were unable to read English well. Individuals with prior cognitive behavioral therapy (including ACT) for chronic pain were also excluded, as such experiences can alter chronic pain acceptance. Those meeting eligibility requirements and providing electronic informed consent were then directed to a 25‐min online survey. Following survey completion, participants were compensated per their agreements with Qualtrics. In accordance with veteran verification procedures used in online surveys (Kelly et al. 2022; Reilly et al. 2022), those reporting veteran status were asked if they had received a DD 214 (Certificate of Release or Discharge from Active Duty form) and, if so, their date of discharge. DD 214 details that were inconsistent with self‐reported years of service or age resulted in exclusion. Participants were also excluded if they failed an attention check (i.e., “To monitor quality, please respond with a two for this item”), completed the survey in less than half of the median completion time, or did not provide complete data for variables of interest. All study procedures and methods were reviewed and approved by the Institutional Review Board at Syracuse University.

Sample size was determined using an a priori power analysis, which was conducted with G*Power (Faul et al. 2007). Consistent with research examining acceptance as a moderator of associations between pain intensity and health‐related behaviors (Elander et al. 2014; Kanzler et al. 2019), a small effect size (*f* ^2^ = 0.02) was estimated (Cohen 1992). With an alpha of 0.05 and power level of 0.80, the necessary sample size was approximately 395.

### 2.2 Measures

#### 2.2.1 Characteristic Pain Intensity

Three items from the Graded Chronic Pain Scale (GCPS; Von Korff et al. 1992) were used to assess characteristic pain intensity (i.e., Characteristic Pain Intensity subscale). Using response options that ranged from 0 (*no pain*) to 10 (*pain as bad as it could be*), participants rated their current, worst, and average pain over the past 3 months. Items were averaged and multiplied by 10, yielding scores ranging from 0 to 100, with higher scores indicating greater pain intensity. Internal consistency of the Characteristic Pain Intensity subscale was good in the current sample (*α* = 0.85).

#### 2.2.2 Chronic Pain Acceptance

The Chronic Pain Acceptance Questionnaire (CPAQ; McCracken et al. 2004) is a 20‐item measure of acceptance of chronic pain. The CPAQ includes two subscales: Pain Willingness (e.g., “I avoid putting myself in situations where my pain might increase.”); and Activity Engagement (e.g., “My life is going well, even though I have chronic pain”). Items are rated on a 7‐point Likert scale ranging from 0 (*never true*) to 6 (*always true*), and Pain Willingness items are reverse scored. Total scores are calculated by summing all items and range from 0 to 120, with higher scores indicating greater pain acceptance. The CPAQ demonstrated excellent internal consistency in the current sample (*α* = 0.90).

#### 2.2.3 Alcohol Use Severity

The Alcohol Use Disorders Identification Test (AUDIT; Saunders et al. 1993) is a 10‐item screening tool designed to identify individuals whose alcohol consumption places them at risk for the development of alcohol use disorder. The AUDIT assesses the quantity and frequency of alcohol use, symptoms of dependence, and alcohol‐related harms. Items are rated on a 0–4 scale, yielding total scores ranging from 0 to 40. Total scores are used as an index of alcohol use severity, with a recommended clinical threshold score of ≥ 8 used to indicate hazardous drinking (Saunders et al. 1993). In the current sample, the internal consistency of the AUDIT total score was good (*α* = 0.89).

#### 2.2.4 Sociodemographic, Military Service, Pain, and Substance Use Characteristics

Participants reported a range of sociodemographic and military service‐related characteristics, including age, race, ethnicity, gender, education, marital status, employment status, income, service branch, service era, and Veterans Health Administration (VHA) connection status. Participants also provided additional pain‐related information, including location, which was assessed using the Michigan Body Map (MBM; Brummett et al. 2016), and pain duration (in months). Current prescription opioid use and cigarette smoking were also assessed with binary (*yes*/*no*) items.

### 2.3 Data Analytic Plan

All analyses were conducted using IBM SPSS Statistics Version 29 and the PROCESS Macro for SPSS (Hayes 2013). Standard diagnostics indicated that model assumptions were met, and no transformations were required. Outliers can negatively affect statistical analyses by increasing error variance and reducing power, decreasing normality, and biasing parameter estimates (Osborne and Overbay 2004). Consistent with methods widely used in regression analyses (Field 2024), cases with standardized residuals exceeding ±3 standard deviations were identified as outliers. Seven cases met this criterion and were excluded from the primary models to minimize potential undue influence. To verify whether conclusions were dependent on this decision, the identical models were also re‐estimated including outliers as a sensitivity analysis.

Following these data preparation procedures, we proceeded with the primary analyses. First, descriptive statistics and zero‐order correlations among sociodemographic, pain, acceptance, and substance‐related variables were calculated. Next, a hierarchical linear regression model was used to test whether CPAQ score moderated the association between Characteristic Pain Intensity and AUDIT total score. Sociodemographic characteristics (i.e., age, race, ethnicity, and gender), opioid use, cigarette smoking, and pain duration were included as covariates based on prior research demonstrating associations with pain intensity and alcohol‐related outcomes (Delker et al. 2016; Mills et al. 2019). Predictors were entered into the model in the following order: Step 1 (covariates); Step 2 (Characteristic Pain Intensity score, CPAQ score); Step 3 (Characteristic Pain Intensity score × CPAQ score interaction). This hierarchical model allowed us to evaluate change in R squared to quantify the relative contribution of each set of predictors. When the interaction term was statistically significant (*p* < 0.05), conditional effects were probed using the PROCESS Macro for SPSS with 10,000 bootstrap samples (Hayes 2013), including estimation of simple slopes of Characteristic Pain Intensity score predicting AUDIT total score at specified levels of CPAQ score (16th, 50th, and 84th percentiles). These moderation analyses were then repeated among a subsample of participants scoring above a clinical threshold for hazardous drinking (i.e., AUDIT total scores ≥ 8).

## 3 Results

### 3.1 Participant Characteristics

Participants included 429 veterans with chronic musculoskeletal pain who endorsed past‐month alcohol use (*M*_age_ = 56.6, SD = 14.7). The sample was predominantly male (75.8%), non‐Hispanic (93.2%), White (73.7%), and married (52.0%). Participants were generally well‐educated, with roughly half completing at least technical school/associate degree, and about 62% reported a total household income greater than $50,000. The most represented service branch was the Army, and approximately half of the sample were current VA healthcare users. Low back was the most commonly endorsed pain location, and more than one‐third of the sample scored above the AUDIT cutoff for hazardous alcohol use. Additional sociodemographic, military, pain, and substance use data are presented in Table 1.

**TABLE 1:** Sociodemographic, military, pain, and substance use characteristics.

	Total sample	Hazardous alcohol use	
		Yes	No
	*n* (%)	*n* (%)	*n* (%)
Gender			
Male	325 (75.8%)	122 (83.0%)	203 (72.0%)
Ethnicity			
Non‐Hispanic	400 (93.2%)	134 (91.2%)	266 (94.3%)
Race			
White	316 (73.7%)	98 (66.7%)	218 (77.3%)
Black or African American	78 (18.2%)	38 (25.9%)	40 (14.2%)
Asian	6 (1.4%)	0 (0%)	6 (2.1%)
American Indian/Alaska Native	5 (1.2%)	3 (2.0%)	2 (0.7%)
Multiracial/Other	24 (5.6%)	8 (5.4%)	16 (5.7%)
Marital status			
Single	74 (17.2%)	43 (29.3%)	31 (11.0%)
Married	223 (52.0%)	57 (38.8%)	166 (58.9%)
Separated/divorced/widowed	132 (30.8%)	47 (32.0%)	85 (30.1%)
Education			
Did not graduate high school	2 (0.5%)	0 (0.0%)	2 (0.7%)
High school graduate or GED	69 (16.1%)	29 (19.7%)	40 (14.2%)
Some college/technical school/associate degree	207 (48.3%)	73 (49.7%)	134 (47.5%)
4‐year college degree	95 (22.1%)	35 (23.8%)	60 (21.3%)
Some school beyond 4‐year college degree	21 (4.9%)	5 (3.4%)	16 (5.7%)
Professional degree (e.g., MD, JD, PhD)	35 (8.2%)	5 (3.4%)	30 (10.6%)
Annual household Income			
Less than $10,000	9 (2.1%)	3 (2.0%)	6 (2.1%)
$10,000–$49,999	155 (36.1%)	62 (42.2%)	93 (33.0%)
$50,000–$100,000	195 (45.5%)	69 (46.9%)	126 (44.7%)
Greater than $100,000	70 (16.3%)	13 (8.8%)	57 (20.2%)
Employment status			
Employed full time	170 (39.6%)	75 (51.0%)	95 (33.7%)
Employed part time	24 (5.6%)	8 (5.4%)	16 (5.7%)
Retired/Disabled	202 (47.1%)	47 (32.0%)	155 (55.0%)
Unemployed/student/homemaker	33 (7.7%)	17 (11.6%)	16 (5.7%)
Service branch^a^			
Army	202 (47.1%)	71 (48.3%)	131 (46.5%)
Navy	96 (22.4%)	38 (25.9%)	58 (20.6%)
Air force	103 (24.0%)	31 (21.1%)	72 (25.5%)
Marine corps	37 (8.6%)	13 (8.8%)	24 (8.5%)
Coast guard	4 (0.9%)	1 (0.7%)	3 (1.1%)
Service era			
Pre‐9/11	292 (68.1%)	79 (53.7%)	213 (75.5%)
Post‐9/11	91 (21.2%)	53 (36.1%)	38 (13.5%)
Both	46 (10.7%)	15 (10.2%)	31 (11.0%)
Pain locations^a^			
Face/jaw/head	52 (12.1%)	22 (15.0%)	30 (10.6%)
Chest/abdomen/pelvis	71 (16.6%)	34 (23.1%)	37 (13.1%)
Neck/upper back/low back	354 (82.5%)	129 (87.8%)	225 (79.8%)
Upper extremities	216 (50.3%)	72 (49.0%)	144 (51.1%)
Hips/groin/buttocks/lower extremities	335 (78.1%)	115 (78.2%)	220 (78.0%)
VA healthcare use	224 (52.2%)	90 (61.2%)	134 (47.5%)
Cigarette smoking	161 (37.5%)	83 (56.5%)	78 (27.7%)
Prescription opioid use	66 (15.4%)	30 (20.4%)	36 (12.8%)
Hazardous drinking	147 (34.3%)		

	*M* (SD)	*M* (SD)	*M* (SD)
Age	56.63 (14.74)	49.98 (13.99)	60.10 (13.93)
Pain duration (in months)	158.04 (134.96)	134.27 (110.73)	170.43 (144.64)
GCPS—Characteristic Pain Intensity	62.01 (16.57)	66.37 (15.63)	59.74 (16.62)
CPAQ—total score	63.57 (18.38)	57.39 (17.86)	66.79 (17.84)
AUDIT—total score	7.52 (7.21)	15.69 (6.63)	3.26 (1.78)

*Note:* Scores of 8+ on the Alcohol Use Disorders Identification Test (AUDIT) indicate hazardous drinking.

Abbreviations: AUDIT, Alcohol Use Disorders Identification Test; CPAQ, Chronic Pain Acceptance Questionnaire; GCPS – Characteristic Pain Intensity, Graded Chronic Pain Scale—Characteristic Pain Intensity subscale.

^a^ Categories are not mutually exclusive.

### 3.2 Zero‐Order Correlations

Zero‐order correlations are presented in Table 2. To summarize, CPAQ score was negatively correlated with Characteristic Pain Intensity and AUDIT total scores, while Characteristic Pain Intensity score was positively correlated with AUDIT total score.

**TABLE 2:** Zero‐order correlations among study variables.

Variable	1	2	3	4	5	6	7	8	9	10
1 Age	—	−0.28**	−0.14**	0.23**	−0.15**	−0.26**	0.24**	−0.32**	0.23**	−0.31**
2 Race		—	0.07	−0.07	0.10*	0.15**	−0.09	0.17**	−0.14**	0.09
3 Ethnicity			—	−0.04	−0.01	−0.07	−0.04	0.05	0.02	0.08
4 Gender				—	−0.08	0.07	0.05	−0.15**	0.15**	0.12*
5 Prescription opioid use					—	0.12*	−0.06	0.24**	−0.18**	0.09
6 Cigarette smoking						—	−0.06	0.26**	−0.21**	0.27**
7 Pain duration							—	< 0.01	0.01	−0.12*
8 GCPS—Characteristic Pain Intensity								—	−0.50**	0.22**
9 CPAQ—total score									—	−0.29**
10 AUDIT—total score										—

*Note:* Race: 0 = White, 1 = minoritized; ethnicity: 0 = non‐Hispanic, 1 = Hispanic; gender: 0 = female, 1 = male; prescription opioid use: 0 = no, 1 = yes; cigarette smoking: 0 = no, 1 = yes.

Abbreviations: AUDIT, Alcohol Use Disorders Identification Test; CPAQ, Chronic Pain Acceptance Questionnaire; GCPS—Characteristic Pain Intensity, Graded Chronic Pain Scale—Characteristic Pain Intensity subscale.

*p < 0.05; **p < 0.01.

### 3.3 Pain Intensity, Chronic Pain Acceptance, and Alcohol Use Severity

**FIGURE 1:**
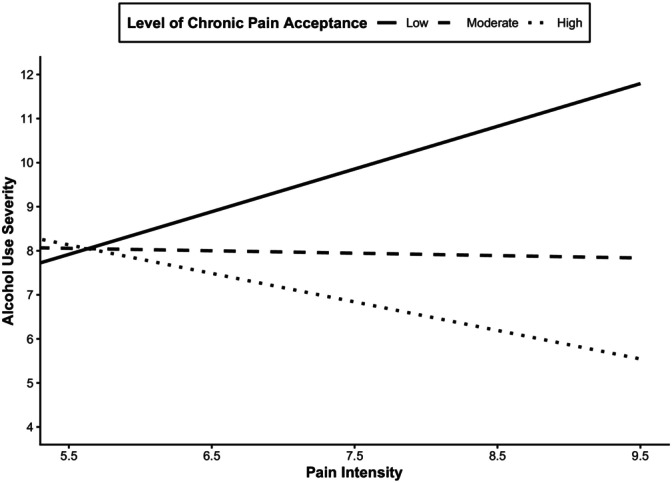
Conditional effects of pain intensity on alcohol use severity at specified levels of chronic pain acceptance. Pain Intensity = Graded Chronic Pain Scale—Characteristic Pain Intensity subscale; Chronic Pain Acceptance = Chronic Pain Acceptance Questionnaire—total score; Alcohol Use Severity = Alcohol Use Disorders Identification Test—total score; Levels of chronic pain acceptance include 16th, 50th, and 84th percentiles (Low, Moderate, and High, respectively); Conditional effects: Low (*b* = 0.063, SE = 0.027, *p* = 0.019, 95% CI [0.010, 0.116]), Moderate (*b* = 0.017, SE = 0.021, *p* = 0.429, 95% CI [−0.025, 0.058]), High (*b* = −0.036, SE = 0.024, *p* = *0*.124, 95% CI [−0.083, 0.010]).

At Step 1 of the hierarchical linear regression, covariates explained a significant portion of variance in AUDIT total score (*R* ^2^ = 0.193, *p* < 0.001; see Table 3). The addition of Characteristic Pain Intensity and CPAQ scores at Step 2 accounted for another 4.7% of the variance (Δ*R* ^2^ = 0.047, *p* < 0.001). At Step 3, the Characteristic Pain Intensity × CPAQ interaction term was statistically significant, further explaining 2.2% of the variance in AUDIT total score (*β* = −0.525, *p* < 0.001; Δ*R* ^2^ = 0.022, *p* < 0.001; Table 3). Conditional effects analysis revealed a significant association between Characteristic Pain Intensity and AUDIT total score at low (*b* = 0.063, SE = 0.027, *p* = 0.019, 95% CI [0.010, 0.116]), but not moderate (*b* = 0.017, SE = 0.021, *p* = 0.429, 95% CI [−0.025, 0.058]) or high (*b* = −0.036, SE = 0.024, *p* = 0.124, 95% CI [−0.083, 0.010]; see Figure 1) levels of CPAQ score. The sensitivity analysis including outliers yielded consistent results across all model steps (Table S1), with conditional effects analysis again indicating a significant association between Characteristic Pain Intensity and AUDIT total score at low (*b* = 0.059, SE = 0.030, *p* = 0.048, 95% CI [0.000, 0.117]), but not moderate (*b* = 0.020, SE = 0.023, *p* = 0.386, 95% CI [−0.025, 0.066]) or high (*b* = −0.023, SE = 0.026, *p* = 0.368, 95% CI [−0.075, 0.028]) levels of CPAQ score. In addition, a sensitivity analysis restricted to participants with high levels of pain‐related disability (pain grades of 3 or 4; *n* = 230) revealed consistent results across all model steps (Table S2), with conditional effects analysis indicating a significant interaction at low (*b* = 0.403, SE = 0.159, *p* = 0.011, 95% CI [0.090, 0.716]), but not moderate (*b* = 0.037, SE = 0.129, *p* = 0.774, 95% CI [−0.218, 0.292]), or high (*b* = −0.174, SE = 0.155, *p* = 0.262, 95% CI [−0.480, 0.131]) levels of CPAQ.

**TABLE 3:** Associations between pain intensity, chronic pain acceptance, and alcohol use severity.

Alcohol use severity^a^	*β*	*t*	*p*	Δ*R* ^2^	*p* for Δ*R* ^2^
Step 1				0.193	< 0.001
Age	−0.325	−6.447	< 0.001		
Race	−0.015	−0.314	0.754		
Ethnicity	0.036	0.796	0.426		
Gender	0.206	4.506	< 0.001		
Cigarette smoking	0.167	3.577	< 0.001		
Prescription opioid use	0.035	0.780	0.436		
Pain duration	−0.048	−1.052	0.293		
Step 2				0.047	< 0.001
Pain intensity^b^	0.009	0.171	0.864		
Chronic pain acceptance^c^	−0.228	−4.516	< 0.001		
Step 3				0.022	< 0.001
Pain intensity × chronic pain acceptance	−0.525	−3.476	< 0.001		

*Note:* Race: 0 = White, 1 = minoritized; ethnicity: 0 = non‐Hispanic, 1 = Hispanic; gender: 0 = female, 1 = male; prescription opioid use: 0 = no, 1 = yes; cigarette smoking: 0 = no, 1 = yes.

^a^ Alcohol Use Disorders Identification Test—total score.

^b^ Graded Chronic Pain Scale—Characteristic Pain Intensity subscale.

^c^ Chronic Pain Acceptance Questionnaire—total score.

### 3.4 Exploratory Subgroup Analysis Among Hazardous Drinkers

**FIGURE 2:**
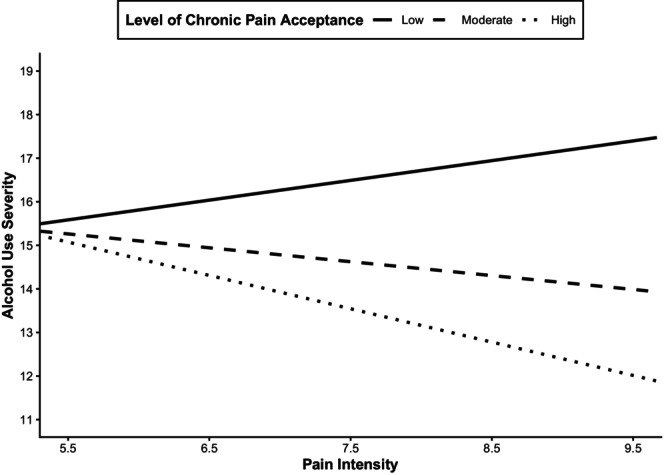
Conditional effects of pain intensity on alcohol use severity at specified levels of chronic pain acceptance among hazardous drinkers. Includes participants who scored above threshold for hazardous drinking (AUDIT total score ≥ 8; *n* = 140); Pain Intensity = Graded Chronic Pain Scale—Characteristic Pain Intensity subscale; Chronic Pain Acceptance = Chronic Pain Acceptance Questionnaire—total score; Alcohol Use Severity = Alcohol Use Disorders Identification Test—total score; Levels of chronic pain acceptance include 16th, 50th, and 84th percentiles (Low, Moderate, and High, respectively); Conditional effects: Low (*b* = 0.090, SE = 0.047, *p* = 0.060, 95% CI [−0.004, 0.183]), Moderate (*b* = 0.029, SE = 0.039, *p* = 0.455, 95% CI [−0.048, 0.107]), High (*b* = −0.016, SE = 0.044, *p* = *0*.713, 95% CI [−0.103, 0.070]).

The hierarchical linear regression among participants who scored above the AUDIT cutoff for hazardous drinking (*n* = 140; 32.6% of the full sample) yielded results that were comparable to those observed in the full sample, with the Characteristic Pain Intensity × CPAQ interaction term remaining significant (*β* = −0.676, *p* = 0.023; Δ*R* ^2^ = 0.034, *p* = 0.023; see Table 4). However, conditional effects analysis revealed no significant association between Characteristic Pain Intensity and AUDIT total score across low (*b* = 0.090, SE = 0.047, *p* = 0.060, 95% CI [−0.004, 0.183]), moderate (*b* = 0.029, SE = 0.039, *p* = 0.455, 95% CI [−0.048, 0.107]), and high (*b* = −0.016, SE *= 0*.044, *p* = 0.713, 95% CI [−0.103, 0.070]; see Figure 2) levels of CPAQ score. The sensitivity analysis including outliers yielded a non‐significant Characteristic Pain Intensity × CPAQ interaction term at Step 3 of the model (*β* = −0.329, *p* = 0.283; Δ*R* ^2^ = 0.008, *p* = 0.283; see Table S2). Therefore, the main effects from Step 2 were examined, where CPAQ score was negatively associated with AUDIT total score (*β* = −0.250, *p* = 0.010) and Characteristic Pain Intensity score was not associated with AUDIT total score (*β* = 0.096, *p* = 0.370; Table S3).

**TABLE 4:** Exploratory subgroup analysis among hazardous drinkers.

Alcohol use severity^a^	*β*	*t*	*p*	Δ*R* ^2^	*p* for Δ*R* ^2^
Step 1				0.032	0.734
Age	−0.177	−1.908	0.059		
Race	−0.053	−0.610	0.543		
Ethnicity	−0.046	−0.522	0.602		
Gender	0.077	0.870	0.386		
Cigarette smoking	−0.031	−0.344	0.732		
Prescription opioid use	0.026	0.294	0.769		
Pain duration	0.019	0.211	0.833		
Step 2				0.113	< 0.001
Pain intensity^b^	0.078	0.722	0.472		
Chronic pain acceptance^c^	−0.305	−3.145	0.002		
Step 3				0.034	0.023
Pain intensity × chronic pain acceptance	−0.676	−2.307	0.023		

*Note:* Includes participants who scored above threshold for hazardous drinking (AUDIT total score ≥ 8; *n* = 140); race: 0 = White, 1 = minoritized; ethnicity: 0 = non‐Hispanic, 1 = Hispanic; gender: 0 = female, 1 = male; prescription opioid use: 0 = no, 1 = yes; cigarette smoking: 0 = no, 1 = yes.

^a^ Alcohol Use Disorders Identification Test—total score.

^b^ Graded Chronic Pain Scale—Characteristic Pain Intensity subscale.

^c^ Chronic Pain Acceptance Questionnaire—total score.

## 4 Discussion

To our knowledge, this is the first study to examine the role of chronic pain acceptance in the relationship between pain intensity and alcohol use severity among veterans with chronic musculoskeletal pain. Consistent with the hypothesis, chronic pain acceptance moderated this relationship such that pain intensity was positively associated with alcohol use severity at low but not moderate or high levels of acceptance. The pain intensity × chronic pain acceptance interaction term accounted for 2.2% of the unique variance in alcohol use severity, a small effect size but one that is typical of interaction effects reported in psychological research (Frazier et al. 2004). Sensitivity analysis indicated that results were robust to the influence of outliers.

Collectively, these findings align with a growing literature implicating chronic pain acceptance in substance use behavior. Research has shown chronic pain acceptance to be negatively associated with opioid use severity and motivation to use opioids for pain coping (Lin et al. 2015; Smit et al. 2023). Additionally, chronic pain acceptance has been found to moderate the relationship between pain intensity and dependence on pain medication, with more intense pain predicting greater dependence at low levels of acceptance but less dependence at high levels of acceptance (Elander et al. 2014). The current results extend this work by indicating that veterans who endorse more intense pain and low levels of chronic pain acceptance may be susceptible to greater alcohol use severity. Like opioids, alcohol can confer acute analgesic effects (Thompson et al. 2017), and drinking behavior among individuals with chronic pain may be motivated, in part, by negative reinforcement processes (McCarthy et al. 2010; Zale et al. 2015). Consistent with the self‐medication hypothesis and conceptualizations of substance use as a means of experiential avoidance (Hayes et al. 1996; Khantzian 1997), veterans who demonstrate less acceptance of their chronic pain may be more likely to use alcohol in attempts to regulate aversive pain states.

Exploratory subgroup analyses suggested chronic pain acceptance may similarly moderate the association between pain intensity and alcohol use severity among veterans scoring above the threshold for hazardous drinking (i.e., AUDIT total scores ≥ 8; *n* = 140). However, despite detecting an interaction, none of the conditional effects were significant, potentially reflecting insufficient statistical power in this subgroup (Hayes 2013). Sensitivity analysis also revealed that the interaction was no longer significant when outliers were included. One possible explanation of this sensitivity is that the reduced sample size of the subgroup limited statistical power, such that the presence of a few extreme cases disproportionately attenuated the interaction effect. Separately, we ran post hoc sensitivity analysis among veterans with high levels of pain‐related disability (GCPS grades 3 or 4; *n* = 230). The pain intensity x chronic pain acceptance interaction remained significant, indicating that the moderating role of chronic pain acceptance was not contingent on high‐impact pain status. Future work with larger and clinically diverse samples will be important to further delineate risk among veterans with hazardous alcohol use.

It is notable that the covariates accounted for 19.3% of the variance in alcohol use severity, representing a meaningful proportion of explained variance in a multifactorial behavioral outcome. Higher alcohol use severity was associated with younger age, male sex, White and Hispanic race/ethnicity, current smoking, and opioid use for pain, consistent with prior findings (National Health Interview Survey 2018; Witkiewitz and Vowles 2018). These findings underscore the potential clinical utility of routinely assessed demographic and behavioral factors for identifying higher‐risk veterans and informing more personalized prevention and intervention strategies. Interestingly, pain duration was negatively associated with alcohol use severity. One plausible explanation is that individuals in their early pain trajectory may be vulnerable to the immediate relief alcohol could provide, which is derived from its capacity to produce acute analgesia, its function as a pain‐coping strategy, and the negatively reinforced avoidance processes described in the experiential avoidance model (Fordyce 1984; Goebel et al. 2011; Riley and King 2009; Thompson et al. 2017). Future work should investigate whether these factors can help delineate clinically meaningful risk subgroups and guide more personalized intervention targets. Furthermore, our recruitment strategy captured veterans both with and without connection to VA health care, a group that is underrepresented in much of the existing literature on chronic pain and alcohol use among veterans. This broader sampling approach may enhance the relevance of findings to veterans who are not engaged in VA care, although patterns of pain, alcohol use, and treatment access may differ from VA‐based clinical samples. Notably, approximately two in three veterans did not receive health care through the VA in 2023 (National Center for Veterans Analysis and Statistics 2025), highlighting the importance of including non‐VA samples for veteran‐related research. However, online recruitment may also have selected for veterans with greater technological access or differing clinical profiles relative to VA‐based samples, and this should be considered when considering generalizability.

A potential clinical implication of the current findings is that chronic pain acceptance may buffer the impact of pain on alcohol use among veterans. This is notable given that drinking for pain management has been linked to escalating consumption over time and other negative health consequences (Brennan et al. 2005; Immonen et al. 2011). From a mechanistic perspective, acceptance‐based interventions may be particularly well‐suited to address co‐occurring chronic pain and hazardous alcohol use, as both conditions are maintained in part by avoidance‐oriented coping strategies. Acceptance and Commitment Therapy (ACT), which is grounded in functional contextualism, emphasizes altering the functional impact of distressing internal experiences (e.g., pain, craving) on behavior rather than eliminating those experiences (Hayes 2004; Hayes et al. 2006). Within this framework, greater pain acceptance may reduce reliance on maladaptive coping behaviors, including alcohol use, in response to pain. This conceptual alignment, along with evidence that acceptance‐based approaches are feasible and relevant for veterans with chronic pain, suggests that ACT‐informed interventions may warrant further evaluation for veterans with comorbid chronic pain and hazardous alcohol use. Importantly, the current findings are cross‐sectional and may inform hypothesis generation for future intervention research.

Several limitations and additional directions for future research should be noted. First, the cross‐sectional design precludes causal inferences. Prospective longitudinal studies will be needed to clarify the temporal precedence of pain intensity, chronic pain acceptance, and alcohol use severity, as well as to examine their interplay in the context of acceptance‐based treatment. Second, the sample was comprised of veterans with chronic musculoskeletal pain and these findings may not generalize to non‐veterans or individuals with other types of chronic pain. For example, associations between chronic pain acceptance and pain‐related adjustment outcomes have been found to vary across pain etiologies (Esteve and Ramírez‐Maestre 2013). Third, given that mental health conditions such as posttraumatic stress disorder, anxiety, and depression are also common among veterans and may independently influence alcohol use (Jakupcak et al. 2010; Tran et al. 2023), future research should examine the role of co‐occurring mental health symptoms. Fourth, the exclusion of individuals with prior CBT experience may limit generalizability. Although this exclusion criterion was used to minimize heterogeneity in baseline levels of pain acceptance, future studies should evaluate whether the present findings hold among veterans with prior CBT experience. Fifth, the current analyses did not examine pain‐related disability in relation to chronic pain acceptance and alcohol use severity. This decision reflected our theoretical focus on acceptance‐related responses to aversive internal stimuli rather than downstream functional outcomes. Nonetheless, pain‐related disability has been linked to both lower chronic pain acceptance and greater alcohol use (White et al. 2024; Zale et al. 2021), and future research should investigate whether chronic pain acceptance moderates relations between pain‐related disability and alcohol use.

In conclusion, the current study offers preliminary insight into relations between pain intensity, chronic pain acceptance, and alcohol use severity. Extending previous work linking chronic pain acceptance to opioid‐related outcomes, these findings suggest that higher levels of pain acceptance may buffer the impact of pain on drinking among veterans. Future research on acceptance‐based interventions for veterans with co‐occurring chronic pain and hazardous alcohol use is warranted.

## Funding

This work was supported by Syracuse University.

## Disclosure

This work was funded by a Syracuse University dissertation fellowship awarded to Kyle M. White. The authors declare no conflicts of interest. Data will be made available upon reasonable request.

## Conflicts of Interest

The authors declare no conflicts of interest.

## Supporting information


**Table S1:** Associations between pain intensity, chronic pain acceptance, and alcohol use severity—with outliers included.
**Table S2:** Associations between pain intensity, chronic pain acceptance, and alcohol use severity—restricted to participants with GCPS Pain Grades of 3 or 4.
**Table S3:** Exploratory subgroup analysis among hazardous drinkers—with outliers included.

## Data Availability

The data that support the findings of this study are available from the corresponding author upon reasonable request.
