# Supplementary material for: Chronic Pain Acceptance Moderates the Association Between Pain Intensity and Alcohol Use Severity Among Veterans With Chronic Musculoskeletal Pain
Source: Alcohol Clin Exp Res (Hoboken). 2026 Jun 12;50(6):e70305. doi: 10.1111/acer.70305 (PMC13262660; doi:10.1111/acer.70305)
Supplement: Supplementary file 1 — Table S1: Associations between pain intensity, chronic pain acceptance, and alcohol use severity—with outliers included. Table S2: Associations between pain intensity, chronic pain acceptance, and alcohol use severity—restricted to participants with GCPS Pain Grades of 3 or 4. Table S3: Exploratory subgroup analysis among hazardous drinkers—with outliers included. [file ACER-50-0-s001.docx]

Supplementary Table 1. *Associations between pain intensity, chronic pain acceptance, and alcohol use* *severity* – *with outliers included*

| **Alcohol Use Severity ^a^** | | | | | |
| --- | --- | --- | --- | --- | --- |
|  | ***β*** | ***t*** | ***p*** | **Δ*R*^2^** | ***p for* Δ*R*^2^** |
| **Step 1** |  |  |  | .168 | <.001 |
| Age | -.277 | -5.445 | <.001 |  |  |
| Race | -.013 | -.275 | .784 |  |  |
| Ethnicity | .061 | 1.358 | .175 |  |  |
| Gender | .176 | 3.825 | <.001 |  |  |
| Cigarette Smoking | .188 | 3.985 | <.001 |  |  |
| Prescription Opioid Use | .034 | .754 | .451 |  |  |
| Pain Duration | -.046 | -.995 | .320 |  |  |
| **Step 2** |  |  |  | .048 | <.001 |
| Pain Intensity ^b^ | .021 | .399 | .690 |  |  |
| Chronic Pain Acceptance ^c^ | -.223 | -4.418 | <.001 |  |  |
| **Step 3** |  |  |  | .013 | .009 |
| Pain Intensity × Chronic Pain Acceptance | -.404 | -2.611 | .009 |  |  |

*Note*. Race: 0 = White, 1 = Minoritized; Ethnicity: 0 = Non-Hispanic, 1 = Hispanic; Gender: 0 = Female, 1 = Male; Prescription Opioid Use: 0 = No, 1 = Yes; Cigarette Smoking: 0 = No, 1 = Yes; ^a^ Alcohol Use Disorders Identification Test – total score; ^b^ Graded Chronic Pain Scale – Characteristic Pain Intensity subscale; ^c^ Chronic Pain Acceptance Questionnaire – total score.

Supplementary Table 2. *Associations between pain intensity, chronic pain acceptance, and alcohol use* *severity* – *restricted to participants with GCPS Pain Grades of 3 or 4.*

| **Alcohol Use Severity ^a^** | | | | | |
| --- | --- | --- | --- | --- | --- |
|  | ***β*** | ***t*** | ***p*** | **Δ*R*^2^** | ***p for* Δ*R*^2^** |
| **Step 1** |  |  |  | .166 | <.001 |
| Age | -.315 | -4.60 | <.001 |  |  |
| Race | -.036 | -.552 | .581 |  |  |
| Ethnicity | .014 | .224 | .823 |  |  |
| Gender | .203 | 3.196 | .001 |  |  |
| Cigarette Smoking | .123 | 1.894 | .060 |  |  |
| Prescription Opioid Use | .020 | .321 | .749 |  |  |
| Pain Duration | -.039 | -.605 | .546 |  |  |
| **Step 2** |  |  |  | .058 | <.001 |
| Pain Intensity ^b^ | .054 | .801 | .424 |  |  |
| Chronic Pain Acceptance ^c^ | -.222 | -3.470 | <.001 |  |  |
| **Step 3** |  |  |  | .034 | .002 |
| Pain Intensity × Chronic Pain Acceptance | -.921 | -3.151 | .002 |  |  |

*Note*. Race: 0 = White, 1 = Minoritized; Ethnicity: 0 = Non-Hispanic, 1 = Hispanic; Gender: 0 = Female, 1 = Male; Prescription Opioid Use: 0 = No, 1 = Yes; Cigarette Smoking: 0 = No, 1 = Yes; ^a^ Alcohol Use Disorders Identification Test – total score; ^b^ Graded Chronic Pain Scale – Characteristic Pain Intensity subscale; ^c^ Chronic Pain Acceptance Questionnaire – total score.

Supplementary Table 3*. Exploratory subgroup analysis among hazardous drinkers – with outliers included*

| **Alcohol Use Severity ^a^** | | | | | |
| --- | --- | --- | --- | --- | --- |
|  | ***β*** | ***t*** | ***p*** | **Δ*R*^2^** | ***p for* Δ*R*^2^** |
| **Step 1** |  |  |  | .009 | .990 |
| Age | -.073 | -.795 | .428 |  |  |
| Race | -.043 | -.496 | .620 |  |  |
| Ethnicity | .026 | .297 | .767 |  |  |
| Gender | .018 | .201 | .841 |  |  |
| Cigarette Smoking | .033 | .380 | .705 |  |  |
| Prescription Opioid Use | .023 | .264 | .792 |  |  |
| Pain Duration | .009 | .105 | .917 |  |  |
| **Step 2** |  |  |  | .087 | .002 |
| Pain Intensity ^b^ | .096 | .900 | .370 |  |  |
| Chronic Pain Acceptance ^c^ | -.250 | -2.610 | .010 |  |  |
| **Step 3** |  |  |  | .008 | .283 |
| Pain Intensity × Chronic Pain Acceptance | -.329 | -1.078 | .283 |  |  |

*Note*. Includes participants who scored above threshold for hazardous drinking (AUDIT total score ≥ 8; *n* = 147); Race: 0 = White, 1 = Minoritized; Ethnicity: 0 = Non-Hispanic, 1 = Hispanic; Gender: 0 = Female, 1 = Male; Prescription Opioid Use: 0 = No, 1 = Yes; Cigarette Smoking: 0 = No, 1 = Yes; ^a^ Alcohol Use Disorders Identification Test – total score; ^b^ Graded Chronic Pain Scale – Characteristic Pain Intensity subscale; ^c^ Chronic Pain Acceptance Questionnaire – total score.
